# Effects of Childhood Multidisciplinary Care and Growth Hormone Treatment on Health Problems in Adults with Prader-Willi Syndrome

**DOI:** 10.3390/jcm10153250

**Published:** 2021-07-23

**Authors:** Karlijn Pellikaan, Anna G. W. Rosenberg, Kirsten Davidse, Anja A. Kattentidt-Mouravieva, Rogier Kersseboom, Anja G. Bos-Roubos, Lionne N. Grootjen, Layla Damen, Sjoerd A. A. van den Berg, Aart J. van der Lely, Anita C. S. Hokken-Koelega, Laura C. G. de Graaff

**Affiliations:** 1 Department of Internal Medicine, Division of Endocrinology, Erasmus Medical Center, University Medical Centre Rotterdam, 3015 GD Rotterdam, The Netherlands; k.pellikaan@erasmusmc.nl (K.P.); a.rosenberg@erasmusmc.nl (A.G.W.R.); k.davidse@erasmusmc.nl (K.D.); s.a.a.vandenberg@erasmusmc.nl (S.A.A.v.d.B.); a.vanderlelij@erasmusmc.nl (A.J.v.d.L.); 2 Department of Internal Medicine, Division of Endocrinology, Center for Adults with Rare Genetic Syndromes, Erasmus Medical Center, University Medical Center Rotterdam, 3015 GD Rotterdam, The Netherlands; 3 Dutch Center of Reference for Prader-Willi Syndrome, 3015 GD Rotterdam, The Netherlands; l.grootjen@kindengroei.nl (L.N.G.); l.damen@erasmusmc.nl (L.D.); a.hokken@erasmusmc.nl (A.C.S.H.-K.); 4 Academic Centre for Growth Disorders, Erasmus Medical Center, University Medical Centre Rotterdam, 3015 GD Rotterdam, The Netherlands; 5 Stichting Zuidwester, 3241 LB Middelharnis, The Netherlands; a.kattentidt@zuidwester.org (A.A.K.-M.); r.kersseboom@zuidwester.org (R.K.); 6 Centre of Excellence for Neuropsychiatry, Vincent van Gogh Institute for Psychiatry, 5803 AC Venray, The Netherlands; aroubos@vvgi.nl; 7 Department of Pediatrics, Subdivision of Endocrinology, Erasmus University Medical Centre—Sophia Children’s Hospital, 3015 GD Rotterdam, The Netherlands; 8 Dutch Growth Research Foundation, 3016 AH Rotterdam, The Netherlands; 9 Department of Clinical Chemistry, Erasmus Medical Center, University Medical Centre Rotterdam, 3015 GD Rotterdam, The Netherlands

**Keywords:** Prader-Willi syndrome, comorbidity, transition to adult care, growth hormone

## Abstract

Prader-Willi syndrome (PWS) is a complex hypothalamic disorder. Features of PWS include hyperphagia, hypotonia, intellectual disability, and pituitary hormone deficiencies. The combination of growth hormone treatment and multidisciplinary care (GHMDc) has greatly improved the health of children with PWS. Little is known about the effects of childhood GHMDc on health outcomes in adulthood. We retrospectively collected clinical data of 109 adults with PWS. Thirty-nine had received GHMDc during childhood and adolescence (GHMDc+ group) and sixty-three had never received growth hormone treatment (GHt) nor multidisciplinary care (GHMDc− group). Our systematic screening revealed fewer undetected health problems in the GHMDc+ group (10%) than in the GHMDc− group (84%). All health problems revealed in the GHMDc+ group had developed between the last visit to the paediatric and the first visit to the adult clinic and/or did not require treatment. Mean BMI and the prevalence of diabetes mellitus type 2 were significantly lower in the GHMDc+ group compared to the GHMDc− group. As all patients who received GHt were treated in a multidisciplinary setting, it is unknown which effects are the result of GHt and which are the result of multidisciplinary care. However, our data clearly show that the combination of both has beneficial effects. Therefore, we recommend continuing GHMDc after patients with PWS have reached adult age.

## 1. Introduction

Prader-Willi syndrome (PWS) is a genetic, neuroendocrine condition caused by the loss of expression of a cluster of maternally imprinted genes on chromosome 15q11–13. This loss can be caused by a paternal deletion (65–75%), a maternal uniparental disomy 15 (mUPD, 20–30%), an imprinting centre defect (ICD, 1–3%), or a paternal chromosomal translocation (0.1%) [1,2,3]. The prevalence of PWS is 1:10,000–1:30,000 [3]. Newborns with PWS usually have severe hypotonia and poor suck resulting in feeding difficulties, which later in infancy switches to excessive eating. Motor and language development is usually delayed, and most patients develop a complex behavioural phenotype during childhood or later in life. Moreover, children and adults with PWS have hypothalamic dysfunction resulting in hyperphagia, pituitary hormone deficiencies, abnormal temperature regulation, and inadequate pain registration [3,4,5,6,7].

Mortality in both children and adults with PWS is high. A study of reported deaths between 1973 and 2015 showed that 25% had died before reaching the age of twenty, 50% before the age of 29, 75% before the age of 42, and 99% before the age of 60 [8]. In most patients, death is the result of a complex interaction between somatic and psychosocial factors [3,9], like hyperphagia [10,11,12], musculoskeletal problems [13,14,15,16,17], low basal metabolic rate (BMR) [18,19,20,21], behavioural challenges [22,23], biochemical anomalies [3,19,24,25,26,27,28,29,30,31,32,33,34], and cardiovascular risk factors (obesity, hypertension, hypercholesterolemia and type 2 diabetes mellitus (DM2)) [3,8,9,35,36,37,38,39,40,41,42]. 

Many of these risk factors can be improved by growth hormone (GH) treatment. For many years, GH treatment in children with PWS has been approved in European countries, the USA, and several other countries worldwide. GH treatment during childhood improves psychomotor development, cognitive functioning, body composition, and LDL-cholesterol values [43,44,45,46,47,48,49,50,51], with few adverse events. The positive effects on body composition are maintained during long-term GH treatment [52,53]. 

GH treatment for children with PWS is often provided in a multidisciplinary (MD) setting and usually involves a paediatric endocrinologist, dietitian, physiotherapist, and a behavioural expert. For adults, MD care is unavailable in many countries. In the Netherlands, adult MD care has only been available since 2015.

The Dutch Centre of reference for Prader-Willi syndrome is treating over 300 patients with PWS, of whom 140 adults. To evaluate the combined effect of GH treatment and MD care (GHMDc), we report the prevalence of physical health problems in three groups: adults with PWS who have received GHMDc from childhood to adulthood (GHMDc+ group); those who never received GHMDc (GHMDc− group) and those who have temporarily received GHMDc during childhood, but GHMDc was discontinued before adulthood (GHMDc± group). 

## 2. Materials and Methods

Ethical review and approval were waived by the Medical Ethics Committee of the Erasmus University Medical Center. This study was performed at the Centre for Adults with Complex Rare Genetic Syndromes (CRGS) at the Erasmus University Medical Centre, Rotterdam, the Netherlands. We retrospectively reviewed the medical files of all adults who visited the MD outpatient clinic of our centre between January 2015 and January 2021 and who underwent our systematic health screening as part of their regular patient care. As described previously (see [9]), systematic screening consists of a structured interview, an extensive physical examination, a medical questionnaire, a review of the medical records, and biochemical measurements. This systematic screening was largely performed during the first visit to the outpatient clinic for CRGS. However, when parameters could not be assessed during the first visit, data from the next available date was used.

As GH treatment was part of MD childhood care, we investigated the combined effect of GH treatment and MD care and were not able to assess the independent effect of GH treatment or MD care.

The GHMDc+ group is defined as the patients who (1) were treated at our reference centre during childhood and adulthood (2) received MD care and GH treatment both during childhood and adolescence, (3) received specialized transitional care before transferring from the paediatric to the adult endocrinology department and (4) still received GH treatment and MD care at the time of this study. The GHMDc− group had never received MD care nor GH treatment before visiting our outpatient clinic for adults with CRGS, neither during childhood nor during adolescence. The patients in the GHMDc± group temporarily received both GH treatment and MD care at our reference centre during childhood but discontinued GHMDc before the transition to adult care. They spontaneously visited the adult endocrinology department several years later, after which the systematic health screening was performed, and MD care was resumed at the outpatient clinic for adults with CRGS. Therefore, patients in the GHMDc± group did not receive GH treatment or MD care between their last appointment at the paediatric endocrinology department and their first appointment at the adult endocrinology department of our reference centre.

MD care during childhood included treatment by a paediatric endocrinologist, a dietitian, a physiotherapist, a nurse practitioner, a physician for people with intellectual disabilities (ID physician), and if indicated, a psychologist. Transitional care included a shared visit with both the paediatric and the adult endocrinologist, followed by alternating visits at the paediatric and adult department until the final transfer to adult endocrinology.

One patient was excluded as he had received GHMDc at our reference centre both during childhood and adolescence but discontinued GH treatment at his own initiative when he reached adulthood. Another patient was excluded because he had received GHMDc during childhood but received GH treatment in another hospital without MD care before transferring to the adult endocrinology department of our reference centre. Eleven patients were excluded because they had received GH treatment during childhood and/or adolescence but did not receive MD care at our reference centre during childhood.

Newly diagnosed (i.e., undetected/undiagnosed) health problems were defined as health problems that had not been diagnosed before referral to our outpatient clinic but were diagnosed during the systematic health screening at our MD outpatient clinic for adults with complex rare genetic syndromes. 

As part of regular patient care, primary caregivers were asked to fill out a medical questionnaire. In this questionnaire, subjective complaints scored on a 5-point Likert scale (1 = rarely or never, 2 = not often and/or not severe, 3 = quite often and/or quite severe, 4 = often and/or severe, 5 = very often and/or very severe). A score of 3 or higher was considered clinically relevant.

### Data Analysis

Statistical analysis was performed using R version 3.6.3. Descriptive statistics for continuous variables are reported as the median and interquartile range (IQR). Dichotomous variables are displayed as count and percentage, *n* (%). As the GHMDc± only contained seven patients, this group was not included in the statistical analysis. We used a chi-squared test to compare living situations, the prevalence of health problems, and subjective complaints between the GHMDc+ and GHMDc− group. To investigate the relationship between the GHMDc+ and the GHMDc− group and BMI and age, we used the Wilcoxon rank sum test. A chi-squared test for trend was used to compare the number of undiagnosed health problems between the GHMDc+ and the GHMDc− group. To investigate the effect of GHMDc on health problems and subjective complaints, the number of newly diagnosed health problems and BMI corrected for age logistic, ordinal, and linear regression models were used and a likelihood ratio test was performed. As this was an exploratory analysis, no correction for multiple testing was performed.

## 3. Results

We included 109 (53 male/56 female) patients who fulfilled the criteria for one of the GHMDc groups. The median age was 28 years (IQR 20–41) (range 18–72 years) and median BMI 29 kg/m^2^ (IQR 26–36). 

Thirty-nine patients had received GHMDc during childhood and adolescence and still received GHMDc at the time of the study (GHMDc+ group). Sixty-three patients had never received GHMDc (GHMDc− group). Seven had temporarily received GHMDc but did not receive GHMDc anymore at the time of the study (GHMDc± group). The median age of the patients in the GHMDc+ group was 20 years (IQR 19–24), compared to 38 years (IQR 31–51) in the GHMDc− group. 

Before referral to our reference centre, 15 adults in the GHMDc− and GHMDc± were treated (only) by a general practitioner, 37 were (only) treated by an ID physician, 8 (only) by an adult endocrinologist, and 8 by an ID physician and an adult endocrinologist. All patients in the GHMDc+ group received MD childhood care at our reference centre before referral to the MD outpatient clinic for adults, see Table 1. 

The prevalence of different health problems is reported in Table 2. In the GHMDc+ group, the BMI and the prevalence of DM2 were significantly lower than in the GHMDc− group, also after correction for age. The median BMI of the GHMDc± group was comparable to the GHMDc− group, while DM2 was rare (*n* = 1, 14%). The systematic screening revealed more undetected health problems in the GHMDc− group (84%) than in the GHMDc+ group (10%). Health problems that were most often newly diagnosed in the GHMDc− group were hypogonadism (for males defined as a serum testosterone concentration 10 nmol/L combined with clinical signs of hypogonadism and for females defined as an absent or irregular menstrual cycle) and vitamin D deficiency (serum vitamin D concentration 50 nmol/L), followed by scoliosis. In the GHMDc+ group newly diagnosed health problems were hypercholesterolemia (*n* = 1), hypothyroidism (*n* = 1) and hypogonadism (*n* = 2). However, all newly diagnosed health problems in the GHMDc+ group had developed between the last visit to the paediatric department and the first visit to the adult outpatient clinic and/or did not require treatment.

Subjective complaints according to GHMDc group are shown in Table 3. Skin picking, food seeking behaviour, daytime sleepiness, temper tantrums, leg edema, snoring, foot complaints, nocturia, fatigue, constipation, thirst, visual complaints, diarrhoea, backache, heartburn/belching, pica (eating non-food items), sexual problems, and difficulty sleeping were more often reported by patients in the GHMDc− group. The sexual problem that was most often reported was an increased libido (often in males receiving testosterone replacement therapy), leading, for example, to masturbation in public or unwanted sexual behaviours towards other patients in the same group home. Feeling cold and stomach aches were more prevalent in the GHMDc+ group. After correction for age, only the differences in prevalence of nocturia (26% in the GHMDc+ group vs. 31% in the GHMDc− group, *p* = 0.04) and snoring (13% in the GHMDc+ group vs. 44% in the GHMDc− group, *p* = 0.01) were significant. When the *p*-value was corrected for age and BMI, snoring was no longer significant (*p* = 0.2).

Characteristics of the patients in the GHMDc± group are shown in Table 4. Six patients discontinued care at the paediatric endocrinology department because they had to be transferred to a different physician after GH treatment was discontinued, as the MD outpatient clinic for adults with PWS did not exist at the time. One patient discontinued care due to personal circumstances. Five of the seven patients showed an increase in BMI during their time without GHMDc, all of at least 5 kg/m^2^. One patient developed hypothyroidism, one DM2, and one hypercholesterolemia. 

## 4. Discussion

We compared health problems in PWS adults who received GH treatment and multidisciplinary care (GHMDc+) versus those who did not (GHMDc−) and found that health outcomes differed significantly between the two groups. 

In our exploratory analysis, GHMDc was associated with a lower prevalence of obesity and DM2. Whereas obesity was a major problem in the GHMDc− group (median BMI 32 kg/m^2^), the GHMDc+ group had a median BMI of 26 kg/m^2^. However, as the GHMDc+ group still received GH treatment and MD care to date, it is unknown whether the beneficial effects were due to the childhood GHMDc, the ongoing GHMDc, or both. As many health problems become more prevalent with age, it is important to note that the mean age of the GHMDc+ group was lower than the GHMDc− group. This can be explained by the fact that the patients in the GHMDc+ group were, by definition, referred directly by a paediatrician during adolescence or early adulthood. Another explanation is that patients were excluded from the GHMDc− group if they had received GH treatment during childhood, which is now standard care for all children with PWS, thus excluding most adolescents. After correction for age, the relationship between GHMDc and BMI and DM2 was still significant, while the relationship between GHMDc and other health problems was not. Both obesity and DM2 are important cardiovascular risk factors. As half of the deaths in PWS are of cardiopulmonary origin [8,54], it is crucial to reduce obesity and DM2 in this vulnerable patient group.

Apart from obesity and DM2, the prevalence of undiagnosed health problems was also higher in the GHMDc− group (84%) and in the GHMDc± group (71%) compared to the GHMDc+ group (10%). This suggests that GHMDc prevents obesity and DM2 in patients with PWS and results in early detection of health problems that would otherwise remain undiagnosed. The fact that the results for the GHMDc± group were similar to the GHMDc− group suggests that the positive effects of GHMDc are only sustained when continued into adulthood. However, this result may be biased as patients with worse health outcomes are probably more likely to seek care from or be referred to our reference centre during adulthood. Additionally, the small size of the GHMDc± group (seven patients) prevents us from drawing any firm conclusions.

Although not significant, the prevalence of hypothyroidism found by our systematic health screening was higher in the GHMDc+ group than in the GHMDc− group. This could be the result of more frequent thyroid hormone measurements during childhood, as part of standard health watch. Additionally, GH treatment can unmask central hypothyroidism in adults with hypopituitarism [55], although this has not been shown in children with PWS [56]. In the GHMDc+ group, hypothyroidism was often mild, without clinical signs.

When we look at the GHMDc± group in more detail, we see that five of the seven patients in the GHMDc± group showed an increase in BMI of at least 5 kg/m^2^ in their time without GHMDc. This resulted in more obesity (*n* = 4, 57%), compared to the GHMDc+ group (*n* = 6, 15%). Additionally, one patient developed hypothyroidism, one DM2, and one hypercholesterolemia, all accompanied by an increase in BMI. It is well known that DM2 and hypercholesterolemia are related to BMI [57,58,59], but also thyroid function can be affected by BMI. Obesity is associated with a higher serum thyroid stimulating hormone (TSH) concentration and a lower serum free thyroxine (free T4) concentration [60,61]. On the other hand, thyroid dysfunction can increase BMI when patients are not accurately treated [61].

Our exploratory analysis for subjective complaints according to GHMDc group showed that adults in the GHMDc+ group reported fewer nocturia and snoring after correction for age. As BMI is an important cause of snoring [62,63], we performed a post hoc analysis. After adjusting the relationship between snoring and GHMDc group for age and BMI, this relationship was no longer significant. This indicates that the lower prevalence of snoring in the GHMDc+ group is mostly caused by the lower BMI. Unfortunately, we had insufficient data to report on the prevalence of sleep apnea (as assessed by polysomnography) in this population. Future research is needed to investigate the relationship between GHMDc and sleep apnea. Nocturia is an important symptom of heart failure and other heart diseases [64], making this an indicator of cardiovascular health. However, more objective assessments of cardiovascular health (e.g., echocardiography) are needed before drawing any firm conclusions. It should be noted that not all patients filled in the questionnaire and that some patients skipped questions for unknown reasons, which could have influenced the results.

There are several aspects of GHMDc that could explain the differences between the GHMDc+ and GHMDc− groups.

### 4.1. The GHMDc+ Group Received GH Treatment

The GHMDc+ group received GH treatment while the GHMDc− group, by definition, did not. GH status and GH treatment have been the subject of extensive research over the last decades. Individuals with PWS display signs and symptoms of GH deficiency, like short stature, small for height hands and feet, increased body fat and low muscle strength, and muscle mass [3]. Although the reported prevalence of GH deficiency in adults with PWS ranges from 0–38% [65,66], these percentages are only a rough estimate as there are no adequate tests to confirm the diagnosis of GH deficiency in patients with PWS [65,67,68]. The GHRH-arginine test does not detect GH deficiency of hypothalamic origin, as the underlying GHRH deficiency is reversed due to the administration of GHRH [69]. The insulin tolerance test (ITT) is able to detect GH deficiency of hypothalamic origin [70], but is often contra-indicated in PWS due to the presence of epilepsy or cardiovascular disease. In addition, placing two indwelling intravenous catheters needed for the ITT is often technically impossible due to disturbed vascularisation and/or obesity [71,72]. Furthermore, hypoglycaemia can be dangerous in patients with intellectual disabilities, as they could be unable to accurately express their symptoms. However, recently, a more easy-to-perform test, e.g., the glucagon test proved encouraging for the detection of GH deficiency in adults with PWS, although this test is also not infallible [73].

In children with PWS, GH treatment is known to improve physical health and cognition and might also improve quality of life (QoL) [45,51,52,74,75,76,77,78], independent of the GH status [50,68]. GH treatment has become standard of care in PWS children, regardless of the presence or absence of GH deficiency [50]. 

In adults with PWS, GH treatment improves body composition (by increasing lean body mass and decreasing fat mass) and muscle strength, and decreases the prevalence of cardiovascular risk factors, even without proven GH deficiency. Furthermore, positive effects on endurance, several aspects of cognition, and quality of life have been reported [79,80,81,82,83,84,85,86,87,88,89]. Despite these beneficial effects, GH treatment is often not reimbursed by healthcare insurance for adults with PWS as GH deficiency cannot be confirmed. However, in the Netherlands, adults that received GH treatment during childhood can continue GH treatment into adulthood.

### 4.2. The GHMDc+ Group Received Structured Transitional Care

The transition from paediatric to adult care is a vulnerable, yet important process. Structured transitional care is important to decrease drop-out [90]. Paepegaey et al. investigated the effect of transitional care in adults with PWS and found that the presence of structured transitional care resulted in a lower BMI [91]. This is in accordance with our study. However, Paepegaey et al. did not find a significant effect on type 2 diabetes mellitus (DM2). In our centre, transitional care includes a shared visit to both the paediatric and the adult endocrinologist, followed by alternating visits at the paediatric and adult department until the final transfer to adult endocrinology. 

### 4.3. The GHMDc+ Group Was Treated in a Centre of Expertise

Due to the rarity of the syndrome, care for patients with PWS should preferably be provided by dedicated physicians with PWS expertise. In our GHMDc− group, most patients were treated by generalists, i.e., ID physicians or general practitioners. Generalists, by definition, have a broad knowledge of common disorders. Although ID physicians are specialized in syndromes, they usually lack the knowledge of internal health problems and are seldom familiar with the diagnostic and therapeutic pitfalls in the screening and treatment of internal and endocrine problems intrinsic to these rare disorders. The high number of undiagnosed and/or untreated health problems revealed by our systematic screening is probably due to referring general practitioners’ unfamiliarity with the internal and endocrine health problems occurring in this syndrome.

### 4.4. The GHMDc+ Group Was Treated in an MD Setting during Childhood

Due to the complexity of the syndrome, care for both children and adults with PWS should preferably be provided by an MD team. Ideally, the MD team consists of a (paediatric) endocrinologist to treat the pituitary hormone deficiencies, a dietitian to provide and guide a diet that compensates for low basal metabolic rate (BMR), a physiotherapist to address musculoskeletal problems and increase muscle mass to optimize BMR and an ID physician, or, if an ID physician is not available, a behavioural therapist to address behavioural issues. Ideally, a clinical neuropsychologist should also be involved, to assess cognitive, adaptive, and behavioural functioning from a developmental, brain, and behavioural perspective. Patients with PWS often have high verbal comprehension abilities compared to their perceptual reasoning abilities [49,92,93]. Therefore, their capacities are often overestimated by caregivers. This can lead to too many responsibilities, which may cause stress, challenging behaviour, and physical problems like hypertension and fatigue. Informing caregivers about the actual capacities of the PWS individual can prevent this overestimation and the associated stress-related somatic and behavioural issues.

### 4.5. The GHMDc+ Group Underwent Systematic Health Screening

Underdiagnosis is a common problem in patients with PWS, due to the high pain threshold, PWS-specific behavioural phenotype, and/or intellectual disability [7]. Health problems can easily be missed when they are not actively screened for. Therefore, regular patient care should include a systematic health watch, including screening for endocrine deficiencies and cardiovascular risk factors. 

### 4.6. Strengths and Limitations

Like every study, our study has strengths and limitations. The strengths of our study are that we had a (for rare disorders) large sample size and that we provide a thorough exploratory analysis of the differences between the GHMDc+ and GHMDc− group. However, the GHMDc± group was small. As GH treatment was part of MD childhood care, we were only able to investigate the combined effect of GH treatment and MD care and could not assess the independent effect of GH treatment or MD care. Another limitation is the limited overlap in age between the GHMDc+ and the GHMDc− group. Therefore, the results of our multivariable analysis should be interpreted with caution. 

## 5. Conclusions

We demonstrated that the combination of growth hormone treatment and multidisciplinary care has beneficial effects in patients with PWS. Therefore, we recommend to continue GHMDc in patients with PWS who have reached adulthood. Unfortunately, this may not always be possible as growth hormone treatment is not available for all adults with PWS. Based on our data on the combined effect of growth hormone treatment and multidisciplinary care, supported by previously reported beneficial effects of GH treatment alone in both children and adults [43,44,45,46,47,48,49,50,51,52,53,68,74,75,76,77,78,79,80,81,82,83,84,85,86,87,88,89], we support the pledge by Hoybye et al. for general approval of growth hormone treatment in adults with PWS [94]. 

## Figures and Tables

**Table 1:** Baseline characteristics of 109 adults with Prader-Willi syndrome according to GHMDc group.

	GHMDc+ ^a^ *n* = 39	GHMDc− ^b^ *n* = 63	GHMDc± ^c^ *n* = 7	Total *n* = 109
Age in years, median (IQR)	20 (19–24)	38 (31–51)	24 (22–26)	28 (20–41)
BMI in kg/m^2^, median (IQR)	26 (22–29)	32 (27–42)	34 (27–37)	29 (26–36)
Obesity (BMI 30 kg/m^2^), *n* (%)	6 (15%)	36 (57%)	4 (57%)	46 (42%)
Overweight (BMI 25–30 kg/m^2^), *n* (%)	17 (44%)	19 (30%)	3 (43%)	39 (36%)
Lean (BMI 19–25 kg/m^2^), *n* (%)	16 (41%)	8 (13%)	0 (0%)	24 (22%)
Male gender, *n* (%)	18 (46%)	33 (52%)	2 (29%)	53 (49%)
Age at diagnosis in years, median (IQR) ^d^	0 (0–2)	9 (3–20)	0 (0–0)	4 (0–13)
Genetic subtype				
Deletion, *n* (%)	20 (51%)	33 (52%)	4 (57%)	57 (52%)
mUPD, *n* (%) ^e^	13 (33%)	25 (40%)	1 (14%)	39 (36%)
ICD, *n* (%)	2 (5%)	0 (0%)	1 (14%)	3 (3%)
Unknown, *n* (%)	4 (10%)	5 (8%)	1 (14%)	10 (9%)
Growth hormone treatment				
Only during childhood, *n* (%)	0 (0%)	0 (0%)	7 (100%)	7 (6%)
Only during adulthood, *n* (%)	0 (0%)	0 (0%)	0 (0%)	0 (0%)
Both, *n* (%)	39 (100%)	0 (0%)	0 (0%)	39 (36%)
Never, *n* (%)	0 (0%)	63 (100%)	0 (0%)	63 (58%)
Current growth hormone treatment, *n* (%)	39 (100%)	0 (0%)	0 (0%)	39 (36%)
Care before referral				
Multidisciplinary childhood care, *n* (%)	39 (100%)	0 (0%)	0 (0%)	39 (36%)
Endocrinologist only, *n* (%)	0 (0%)	8 (13%)	0 (0%)	8 (7%)
ID-physician only, *n* (%)	0 (0%)	34 (54%)	3 (43%)	37 (34%)
Endocrinologist and ID-physician, *n* (%)	0 (0%)	5 (8%)	3 (43%)	8 (7%)
General practitioner only, *n* (%)	0 (0%)	14 (22%)	1 (14%)	15 (14%)
Unknown, *n* (%)	0 (0%)	2 (3%)	0 (0%)	2 (2%)
Use of hydrocortisone				
Daily, *n* (%)	0 (0%)	2 (3%)	0 (0%)	2 (2%)
During physical or psychological stress, *n* (%)	34 (87%) ^f^	8 (13%)	2 (29%)	44 (40%)
Living situation				
With family, *n* (%)	19 (49%)	6 (10%) ^g^	4 (57%)	29 (27%)
In a specialized PWS group home, *n* (%)	13 (33%)	8 (13%)	0 (0%)	21 (19%)
In a non-specialized facility, *n* (%)	7 (18%)	49 (78%)	3 (43%)	59 (54%)
Scholar level				
Secondary vocational education, *n* (%)	2 (5%)	2 (3%)	0 (0%)	4 (4%)
Pre-vocational secondary education, *n* (%)	3 (8%)	0 (0%)	0 (0%)	3 (3%)
Special education, *n* (%)	31 (79%)	40 (64%)	5 (71%)	76 (70%)
No education, *n* (%)	1 (3%)	4 (6%)	0 (0%)	5 (5%)
Unknown, *n* (%)	2 (5%)	17 (27%)	2 (29%)	21 (19%)
Mutism, *n* (%)	0 (0%)	3 (5%)	0 (0%)	3 (3%)
Relationship status				
In a relationship with sexual intercourse, *n* (%)	2 (5%)	5 (8%)	0 (0%)	7 (6%)
In a relationship without sexual intercourse, *n* (%)	5 (13%)	9 (14%)	1 (14%)	15 (14%)
Not in a relationship, *n* (%)	28 (72%)	40 (64%)	4 (57%)	72 (66%)
Unknown, *n* (%)	4 (10%)	9 (14%)	2 (28%)	15 (14%)

Abbreviations: body mass index (BMI), imprinting centre defect (ICD), physician specialized in intellectual disabilities (ID-physician), interquartile range (IQR), maternal uniparental disomy (mUPD), Prader-Willi syndrome (PWS). ^a^ The GHMDc+ group is defined as the patients who received growth hormone treatment and multidisciplinary care both during childhood and adolescence and received transitional care. ^b^ The GHMDc− group had not received growth hormone treatment or multidisciplinary care during childhood or adolescence. ^c^ The GHMDc± group had received growth hormone treatment and multidisciplinary care during childhood, but not continuously until transfer. ^d^ Only known for 66 patients. ^e^ In 13 patients with an mUPD, the parents were not available for genetic testing. Therefore, mUPD is the most likely genotype, but an ICD could not be ruled out in these patients. ^f^ Many patients in the GHMDc+ group received hydrocortisone during physical or psychological stress as part of regular childhood care, according to the guidelines for the treatment of children with PWS. ^g^ *p*-value for living situation in the GHMDc+ compared to the GHMDc− group is 0.001.

**Table 2:** Health problems according to GHMDc group.

	Missing	GHMDc+ ^a^ *n* = 39	GHMDc− ^b^ *n* = 63	*p*-Value	*p*-Value Corr. for Age ^c^	GHMDc± ^d^ *n* = 7
Age in years, median (IQR)	0	20 (19–24)	38 (31–51)	0.001	NA	24 (22–26)
BMI in kg/m^2^, median (IQR)	0	26 (22–29)	32 (27–42)	0.001	0.001	34 (27–37)
Newly diagnosed health problems ^e^						
At least one		4 (10%) ^f^	53 (84%)			5 (71%)
At least two		0 (0%)	26 (41%)	0.001	0.001	3 (43%)
Three or more		0 (0%)	9 (14%)			0 (0%)
Hypogonadism						
Male (*n* = 53)	1	17 (94%)	32 (100%)	0.2	0.1	2 (100%)
*Of whom treated*		*14* (*82*%)	*6* (*19*%)			*1* (*50*%)
Female (*n* = 56)	13 ^g^	15 (94%)	21 (91%)	0.8	0.5	4 (100%)
*Of whom treated*		*14* (*93*%)	*5* (*24*%)			*1* (*25*%)
Hypothyroidism	0	8 (21%)	7 (11%)	0.2	0.1	1 (14%)
*Of whom treated*		*7* (*88*%) ^h^	*7* (*100*%)			*0* (*0*%)
Diabetes mellitus type 2	3	0 (0%)	16 (27%)	0.001	0.005	1 (14%)
*Of whom treated*		*NA*	*12* (*75*%)			*0* (*0*%)
Hypertension	3	2 (5%)	17 (27%)	0.005	0.8	1 (20%)
*Of whom treated*		*1* (*50*%) ^i^	*13* (*76*%)			*1* (*100*%)
Hypercholesterolemia	2	3 (8%)	18 (30%)	0.01	0.2	1 (14%)
*Of whom treated*		*0* (*0*%) ^j^	*11* (*61*%)			*0* (*0*%)
Scoliosis	4	28 (72%)	42 (71%)	0.9	0.2	7 (100%)
Vitamin D deficiency	42 ^k^	25 (71%)	24 (92%)	NA ^L^	NA ^L^	5 (83%)

Abbreviations: body mass index (BMI), interquartile range (IQR). Data are presented as *n* (%), unless otherwise specified. “Of whom treated” refers to how many patients were treated before undergoing our systematic health screening. Only *p*-values for GHMDc+ vs. GHMDc− are calculated. ^a^ The GHMDc+ group is defined as the patients who received growth hormone treatment and multidisciplinary care both during childhood and adolescence and received transitional care. ^b^ The GHMDc− group had not received growth hormone treatment or multidisciplinary care during childhood or adolescence. ^c^ *p*-value corrected for age using regression models. ^d^ The GHMDc± group had received growth hormone treatment and multidisciplinary care during childhood, but not continuously until transfer. ^e^ Newly diagnosed health problems are: hypogonadism, hypothyroidism, type 2 diabetes mellitus, hypertension, hypercholesterolemia, scoliosis and vitamin D deficiency. Newly diagnosed health problems were health problems that had not been diagnosed before referral to our outpatient clinic, but were diagnosed during the systematic health screening at our multidisciplinary outpatient clinic for adults with complex rare genetic syndromes. ^f^ One patient had newly diagnosed hypercholesterolemia, which had developed between the last visit to the paediatric endocrinologist (where LDL-cholesterol was normal) and the first visit to the adult endocrinologist. One patient had newly diagnosed hypothyroidism with a fluctuating free thyroxin level, which was not treated as discussed with the patient. Two patients had newly diagnosed hypogonadism, of whom one had developed hypogonadism between the last visit to the paediatric endocrinologist (where testosterone was normal) and the first visit to the adult endocrinologist and the other one could not be treated due to severe behavioural problems. ^g^ (Caregivers of) 13 female patients did not recall whether they had had a normal menstrual cycle before the start of oral contraceptives or before reaching menopausal age. ^h^ One patient with hypothyroidism with a fluctuating free thyroxin level, which was not treated as discussed with the patient. ^i^ One patient had untreated moderate hypertension, which was being monitored. ^j^ Treatment not indicated based on the Dutch cardiovascular risk guidelines. ^k^ In 2 patients vitamin D was not measured and 40 patients used vitamin D supplementation before the screening, but it was unknown whether they had low vitamin D values before the start of vitamin D supplementation. ^L^ A *p*-value could not be calculated due to selective missing values.

**Table 3:** Subjective complaints.

	Observations	GHMDc+ ^a^ *n* *=* 39	Observations	GHMDc− ^b^ *n* *=* 63	*p*-Value	*p*-Value Corr. for Age ^c^
Skin picking	31	15 (48%)	49	32 (65%)	0.1	0.5
Food seeking behaviour	30	9 (30%)	49	27 (55%)	0.03	0.5
Daytime sleepiness	31	9 (29%)	51	29 (57%)	0.01	0.3
Temper tantrums	30	10 (33%)	51	25 (49%)	0.2	0.9
Leg edema	30	3 (10%)	51	25 (49%)	0.001	0.2
Snoring	31	4 (13%)	52	23 (44%)	0.003	0.01 ^d^
Foot complaints	31	8 (26%)	50	19 (38%)	0.3	0.7
Nocturia	31	8 (26%)	49	15 (31%)	0.6	0.04
Fatigue	30	6 (20%)	49	14 (29%)	0.4	0.5
Feeling cold	29	11 (38%)	50	6 (12%)	0.007	NA ^e^
Constipation	32	4 (13%)	51	13 (26%)	0.2	NA ^e^
Thirst	30	6 (20%)	47	12 (26%)	0.6	NA ^e^
Visual complaints	30	5 (17%)	48	10 (21%)	0.6	NA ^e^
Stomach ache	32	5 (16%)	49	6 (12%)	0.7	NA ^e^
Diarrhoea	32	2 (6%)	50	9 (18%)	0.1	NA ^e^
Backache	30	3 (10%)	49	9 (18%)	0.3	NA ^e^
Heartburn/belching	30	2 (7%)	51	11 (22%)	0.1	NA ^e^
Pica (eating non-food items)	30	1 (3%)	48	7 (15%)	0.1	NA ^e^
Sexual problems	31	1 (3%)	48	6 (13%)	0.2	NA ^e^
Difficulty sleeping	29	1 (3%)	50	7 (14%)	0.1	NA ^e^

Data are presented as *n* (%). Complaints are scored as present when the caregivers indicated a score of 3 or higher on a 5-point Likertscale. ^a^ The GHMDc+ group is defined as the patients who received growth hormone treatment and multidisciplinary care both during childhood and adolescence and received transitional care. ^b^ The GHMDc− group had not received growth hormone treatment or multidisciplinary care during childhood or adolescence. ^c^ *p*-value corrected for age using logistic regression models. ^d^ Post hoc analysis: *p*-value after correction for age and BMI was 0.2. ^e^ *p*-value was not calculated as there were too few patients with the outcome to fit the model.

**Table 4:** Characteristics GHMDc± group.

	GHMDc± Group (*n* = 7)	
Male/female	2/5	
Total duration of growth hormone treatment, median (IQR)	4.7 (2.7–8.0)	
	**Last visit paediatric endocrinologist**	**First visit adult endocrinologist**
Age in years, median (IQR)	15 (14–18)	24 (22–26)
BMI in kg/m^2^, median (IQR)	28 (27–33)	34 (27–36)

## Data Availability

The datasets generated during and/or analyzed during the current study are not publicly available but are available from the corresponding author on reasonable request.
